# Deciphering the structure of *Arabidopsis thaliana* 5-*enol*-pyruvyl-shikimate-3-phosphate synthase: An essential step toward the discovery of novel inhibitors to supersede glyphosate

**DOI:** 10.1016/j.csbj.2022.03.020

**Published:** 2022-03-24

**Authors:** Milosz Ruszkowski, Giuseppe Forlani

**Affiliations:** a Department of Structural Biology of Eukaryotes, Institute of Bioorganic Chemistry, Polish Academy of Sciences, Poznan, Poland; b Synchrotron Radiation Research Section of MCL, National Cancer Institute, Argonne, IL, USA; c Department of Life Science and Biotechnology, University of Ferrara, Ferrara, Italy

**Keywords:** Enzyme features, EPSP synthase, Glyphosate, Protein dynamics, Conformational change

## Abstract

Glyphosate interferes with plant aromatic metabolism through the inhibition of 5-*enol*-pyruvyl-shikimate-3-phosphate (EPSP) synthase [EPSPS, EC 2.5.1.19]. For this reason, EPSPS has been extensively studied in a vast array of organisms. This notwithstanding, up to date, the crystal structure of the protein has been solved exclusively in a few prokaryotes, while that of the plant enzyme has been only deduced *in silico* by similarity. This study aimed at determining the structure of EPSPS from the plant model species *Arabidopsis thaliana*, which has been cloned, heterologously expressed and affinity-purified. The kinetic properties of the enzyme have been determined, as well as its susceptibility to the inhibition brought about by glyphosate. The crystal structure of the protein has been resolved at high resolution (1.4 Å), showing open conformation of the enzyme, which is the state ready for substrate/inhibitor binding. This provides a framework for the structure-based design of novel EPSPS inhibitors. Surface regions near the active-site cleft entrance or at the interdomain hinge appear promising for inhibitor selectivity, while bound chloride near the active site is a potential placeholder for anionic moieties of future herbicides.

## Graphical abstract

**Figure:**
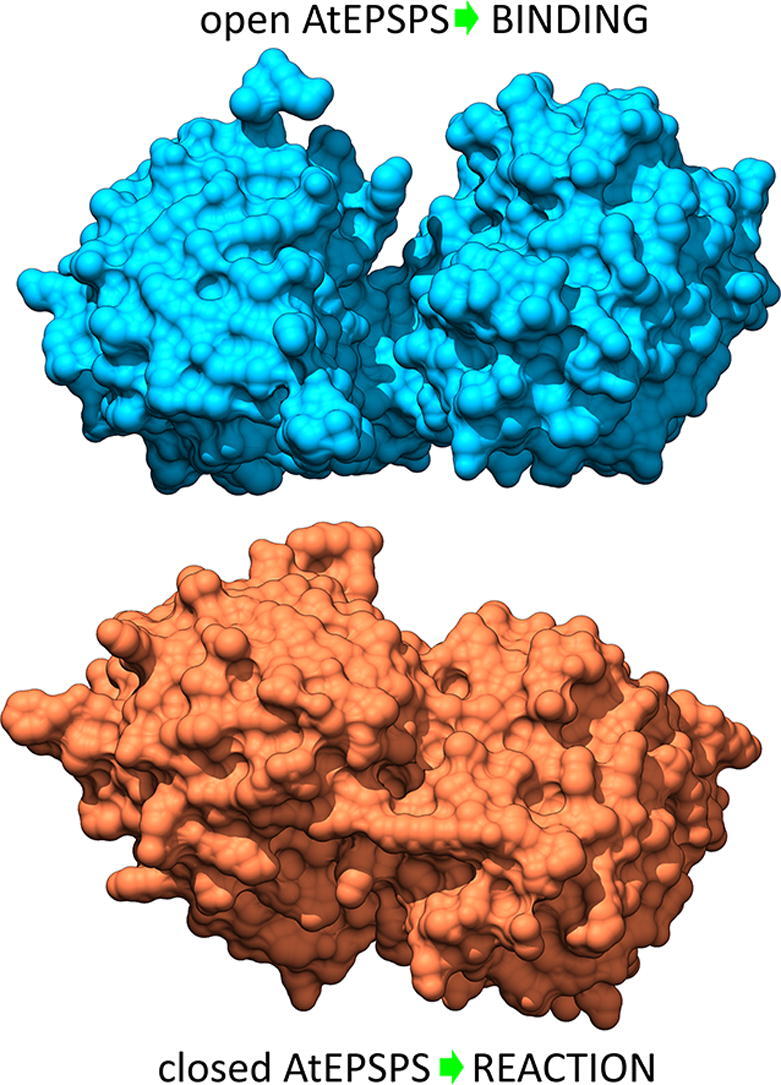

## 1 Introduction

Weed control represents a basic factor for the successful cultivation of crops. Nowadays, crop protection is achieved by the use of several dozens of selective herbicides targeting some peculiar aspects of plant metabolism, while crop tolerance is usually based on the presence/induction of detoxifying enzymes [1]. However, the application of the same active principle for consecutive years gradually led to the selection of spontaneous weed mutants owing tolerance to changes in the structure of the herbicide target [2]. The rapid diffusion of such herbicide-tolerant biotypes threatens food production around the world [3]. Moreover, at the present rate, an increase in the atmospheric CO_2_ level will probably escalate the problem [4].

Currently, the most common herbicide is glyphosate (*N*-[phosphonomethyl]glycine), which is the active ingredient of Roundup produced by Monsanto/Bayer [5]. Glyphosate is a non-selective post-emergence herbicide whose utilization was initially limited because of its inability to distinguish crops from weeds. However, the availability of genetically modified crops that are tolerant because of the presence of genes coding for either a resistant form of the target enzyme or a glyphosate-metabolizing protein made it the most successful herbicide ever [6], [7], [8], [9], [10], [11]. In USA, 80% of corn and 93% of soybean production are based on Roundup-Ready technology, *i.e.* glyphosate-resistant seeds [12]. As the soil microflora rapidly degrades it to CO_2_, ammonia and inorganic phosphate, glyphosate is also considered an environmentally friendly herbicide [13].

However, increasing concern has been recently raised on the alleged carcinogenic activity of glyphosate. The debate began in 2015, when the World Health Organization declared glyphosate as “probably carcinogenic to humans”, based on the assessment by the International Agency for Research on Cancer (IARC) [14]. In 2018, eighteen European Union countries renewed the license for glyphosate use for five years, but the future of this herbicide is uncertain from the legislative perspective. Therefore, significant effort should be invested in finding new active principles to replace it in time [6], [15].

**Fig. 1:**
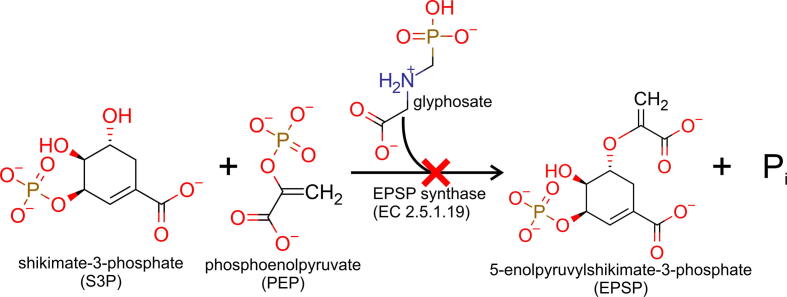
Scheme of the reaction catalyzed by EPSPS. The enzyme is inhibited by the phosphonate herbicide glyphosate with a mechanism of competitive type with respect to PEP. Adapted from Funke et al. [52], with modifications.

Glyphosate acts by inhibiting the activity of 5-*enol*-pyruvyl-shikimate-3-phosphate (EPSP) synthase [EPSPS, EC 2.5.1.19], the enzyme that catalyzes the sixth step in the shikimate pathway (Fig. 1), which provides carbon skeletons for the synthesis of the three aromatic amino acids phenylalanine, tyrosine and tryptophan [5], [16]. In plants, these amino acids are also the entry points toward the synthesis of a plethora of secondary metabolites playing a pivotal role in the interactions with both allies and foes, as well as in the plant defense response to some abiotic stress conditions [17]. The occurrence of this complex metabolic network explains the remarkable and rapid phytotoxicity of glyphosate and makes EPSPS (and other enzymes in the shikimate pathway) an attractive target for the development of new herbicides [18]. For instance, 7-deoxy-sedoheptulose has been recently discovered as an inhibitor of 3-dehydroquinate synthase (EC 4.2.3.4, the enzyme catalyzing the second step in the pre-chorismate pathway) with herbicidal efficacy *in vivo* [19]. Nevertheless, after the identification of EPSPS as the main target of glyphosate, hundreds of papers investigated the properties of this enzyme, mainly aiming at the identification of herbicide-tolerant variants. The exposure of plants to lethal concentrations of glyphosate led to the selection of mutants owing tolerance to EPSPS overexpression, whereas only a few resistant target enzymes were identified [20], [21].

Contrary to other herbicides, for which resistant biotypes appeared rapidly after their introduction in the field, for quite a long time glyphosate resistance was not reported among weeds [22], [23], [24]. This may be due to the characteristics of glyphosate inhibition; glyphosate is competitive to phospho*enol*pyruvate (PEP) and uncompetitive to shikimate-3-phosphate (S3P) [25]. Consequently, most mutations conferring tolerance to glyphosate also negatively impact the affinity for PEP, thereby reducing the enzyme's catalytic efficiency and possibly the fitness of the individuals bearing them [21], [26]. However, *in vitro* selection and the isolation of strains from the soil of glyphosate-producing plants allowed the identification of some mutant enzymes coupling herbicide tolerance and the maintenance of catalytic efficiency, which were subsequently used to obtain glyphosate-resistant crops [7].

Despite the number of studies, the structure of the protein has not been described to date for any plant EPSPS. The only experimental structures of the enzyme are from prokaryotes, among which are *Escherichia coli*, *Coxiella burnetii*, *Vibrio cholerae*, *Mycobacterium tuberculosis*, *Streptococcus pneumoniae* and the glyphosate-resistant strain *Agrobacterium* sp. CP4 (https://www.rcsb.org/). The availability of the structure of a target enzyme allows computer-aided analysis of inhibitor binding, leading to the design of new putative inhibitors by molecular modeling techniques, such as virtual screening through docking. Implementation of such an approach and the subsequent evaluation of the effectiveness of the designed compounds can greatly improve the inhibitory potential of a lead substance, while mitigating the cost of its development. Indeed, some other compounds able to interfere with the activity of EPSPS have been reported, yet their effectiveness against the plant enzyme could be optimized employing this approach [27], [28], [29], [30]. However, in several instances, similar attempts failed when the unavailability of the structure of the plant target forced docking analysis to be performed on an enzyme from non-plant sources. This was, for instance, the case of bisphosphonate inhibitors of *δ*^1^-pyrroline-5-carboxylate reductase (P5CR, EC 1.5.1.2), the enzyme that catalyzes the last common step in both the routes leading to proline synthesis in plants [31]. Inhibitors, designed on the basis of the structure of a bacterial enzyme, were much more effective against bacterial and human P5CR than against the plant counterparts [32], [33], [34], [35].

Here we report on the structure of EPSPS from the plant model species *Arabidopsis thaliana*. Protein structure has been determined in an open conformation at high resolution and compared to the enzymes from other sources, allowing the identification of both similarities and differences. Moreover, a comparison of the experimental open structure and the *in silico* model of the closed form provided insights into the enzyme dynamics. Results are expected to provide a sound basis for the future design of novel inhibitors targeting the shikimate pathway. The first structure of a plant EPSPS enzyme also provides a framework that can be used to identify and design glyphosate-resistant variants of the enzyme.

## 2 Results and discussion

### 2.1 Functional features of *A. thaliana* EPSPS

**Fig. 2:**
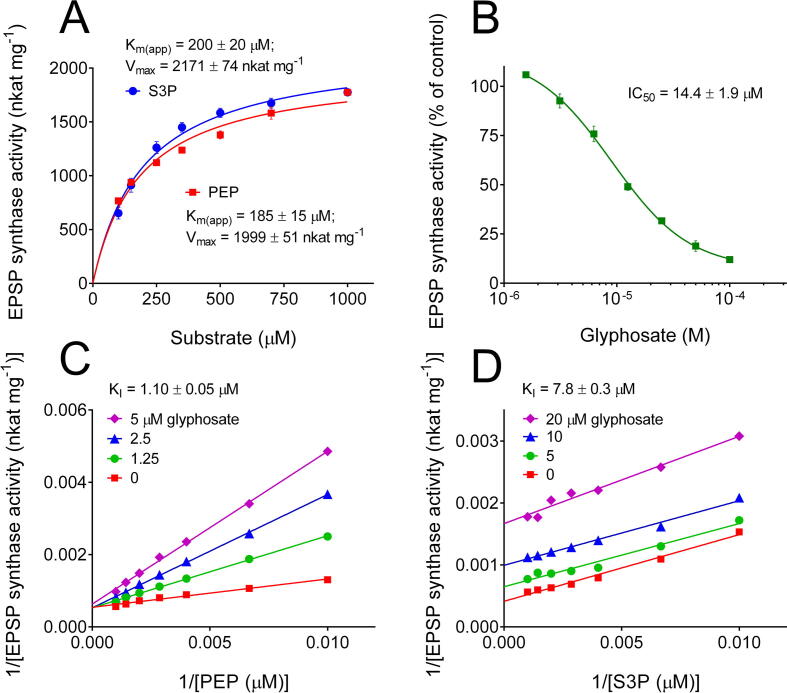
Kinetic analysis of *A. thaliana* EPSPS. The purified enzyme was assayed at varying concentration of a substrate while maintaining the other at 1 mM, allowing the determination of apparent affinity constants and V_max_ values (Panel A). The addition of glyphosate to the reaction mixture was found to progressively inhibit the enzyme activity at concentrations exceeding 2 μM (Panel B). To evaluate inhibition constants, the activity was measured in the presence of increasing levels of glyphosate at varying the concentration of PEP (Panel C) or S3P (Panel D). All values are means of 15 replicates, obtained in 5 independent experiments carried out with three different enzyme preparations.

Heterologous expression of *A. thaliana* EPSPS (AtEPSPS, Uniprot ID: P05466) in *E. coli* and affinity purification yielded an active enzyme, with a specific activity of 1775 ± 158 nkat mg^−1^ protein, a value notably higher than those reported for other plant enzymes (192–750 nkat mg^−1^ protein) and slightly higher than those measured in the case of the two isozymes resolved in maize (1000 and 1600 nkat mg^−1^ protein) [36], [37], [38], [39], [40], [41]. Enzyme stability strictly required the addition of glycerol, EDTA and a reducing agent (dithiothreitol) to the extraction buffer. In their absence, more than 50% activity was lost following 24 h storage at 4 °C. With such additions, more than 80% activity was retained after one week at 4 °C. The functional properties of AtEPSPS, never described before, were carefully determined. The purified protein showed apparent affinity constants (K_M(app)_) of 185 and 200 μM for PEP and S3P, respectively (Fig. 2A). These values are significantly higher than those reported for the enzyme from other plant sources, ranging from 10 to 80 μM [25], [36], [37], [39], [40]. However, in all cases, K_M_s for the two substrates were very similar, a feature that seems a peculiarity of EPSPS. Maximal activity under saturating conditions ranged from 2000 to 2100 nkat mg^−1^ protein, which corresponds to *k*_cat_ of about 95–100 catalytic events s^−1^. The addition of micromolar levels of glyphosate to the standard assay mixture, in which the two substrates are present at 1 mM each, reduced the catalytic rate of AtEPSPS progressively, with a concentration inhibiting activity by 50% (IC_50_) of about 14 μM (Fig. 2B). However, because IC_50_ is influenced by the amount of enzyme, and glyphosate acts with two different mechanisms with respect to either substrate, a proper kinetic analysis was performed by varying the concentration of a single substrate in the presence of increasing levels of the inhibitor. Lines convergent to the y-axis in the Lineweaver-Burk plot confirmed a mechanism of competitive type with respect to PEP, with a K_I_ value of about 1 μM (Fig. 2C). On the contrary, parallel lines were suggestive of a mechanism of uncompetitive type with respect to S3P, with a K_I_ of about 8 μM (Fig. 2D). The latter value is very similar to those reported for other plant EPSPSs (11–18 μM) [38], [39], whereas the K_I_ with respect to PEP is similar to that of the enzyme from *Nicotiana sylvestris* (1.25 μM) but higher than those of most other plant enzymes characterized so far (0.08–0.32 μM), a fact that is consistent with the slightly lower affinity for the substrate [25], [36], [37], [38], [40]. The K_I_/K_M_ ratio, equal to 0.006, places AtEPSPS among the most sensitive enzymes described to date.

### 2.2 Overall properties of the AtEPSPS structure

AtEPSPS crystallized in the *I*422 space group with a single protein molecule in the asymmetric unit. The high-resolution (1.4 Å) electron density maps were of excellent quality and allowed to trace all residues starting from Lys77 until the C-terminal His520. The only region whose definition in the maps was poor was Leu249-Ser252, for which only the main chain was visible. Except for the protein residues, the final model contains 478 water molecules, one magnesium cation and one chloride anion.

Based on both the size-exclusion retention volume (not shown) and the analysis of intermolecular contacts in the crystal lattice by the PDBePISA webserver [42], AtEPSPS is a monomeric protein (Mw = 47.6 kDa for residues 77–520). With the only exception of a dimeric form of the enzyme found in most cyanobacteria, the monomeric state is universal among homologous EPSPS proteins [25], [43], [44]. However, unlike most EPSPS enzymes characterized to date, AtEPSPS crystallized in an open conformation. Attempts were made to obtain complex structures with S3P, shikimate, and glyphosate. However, in each case, the open conformation without bound ligands was found in the structures. It is also unlikely that the 76 N-terminal residues impact conformational changes or substrates binding due to the high sequence variability within this region in plant species (not shown).

**Fig. 3:**
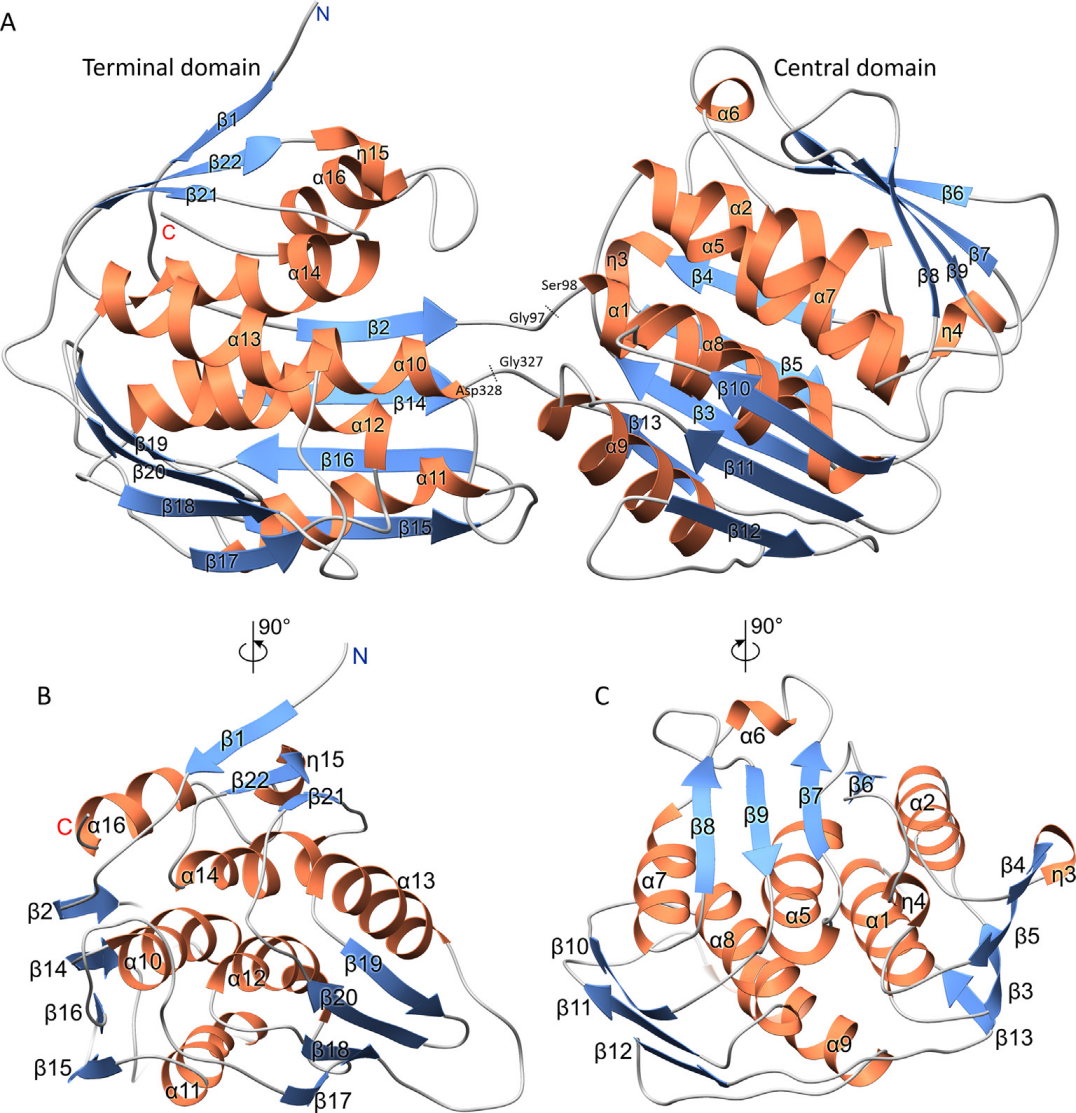
The overall structure of AtEPSPS. Secondary structure elements are colored coral (helices), blue (strands) and gray (coil). Boundaries of the terminal and central domains are indicated in panel A, whereas panels B and C present rotated views over the respective domains. 3_10_ helices are labeled as “η”. Approximate three-fold symmetry axes are normal to the viewing plane in panels B and C. (For interpretation of the references to color in this figure legend, the reader is referred to the web version of this article.)

AtEPSPS folds into two easily distinguishable domains (Fig. 3). In this paper, they are referred to as the terminal domain, containing residues 77–97 and 328–520, and the central domain encompassing the remaining residues 98–327. Structures of the domains are somewhat similar to each other as both are made up of three core α-helices surrounded by three 3–4-stranded mixed β-sheets intertwined by helices. Within each domain, the principal α-helices and β-sheets lie roughly parallel to each other, except for the short helices (Fig. 3). Finally, an approximate three-fold symmetry axis, parallel to the secondary structure elements, multiplies the folding units of βαβαββ topology (Fig. 3B,C).

### 2.3 The active site of AtEPSPS

**Fig. 4:**
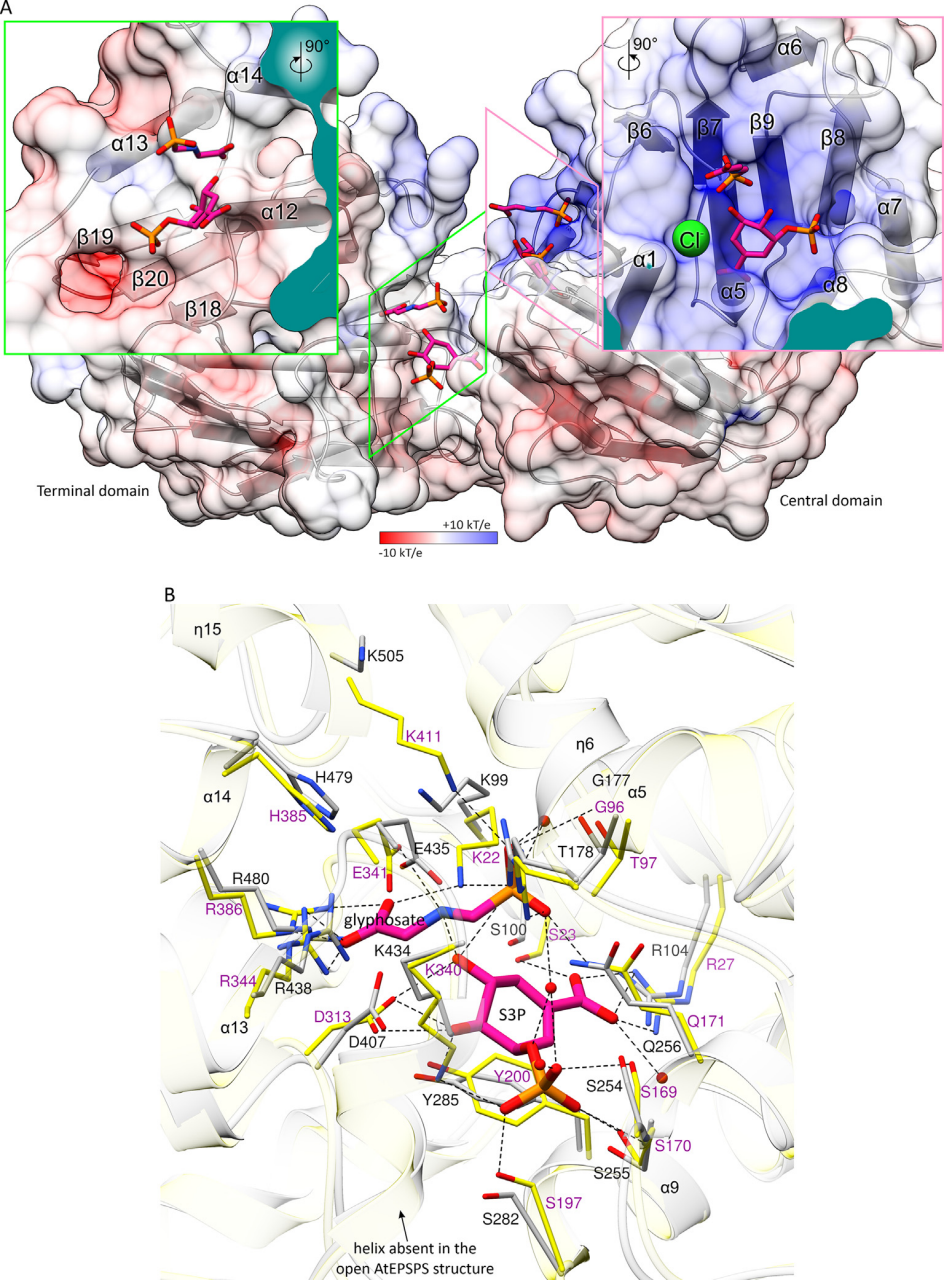
Features of the active site of AtEPSPS. Panel A presents structure in surface representation with electrostatic potential distribution according to the key (bottom). Secondary structure elements are shown as pipes and planks. Mapping of the ligand-binding surface areas onto AtEPSPS in open conformation was performed by superimposing domains of the EcEPSPS structure in complex with S3P and glyphosate (PDB ID: 1g6s [45]). This way, the binding sites were mapped independently for the terminal and central domain as illustrated in the insets. This views likely represent the *in vivo* scenario whereby ligand binding occurs prior to the enzyme closing. The chloride atom (green sphere) observed in the presented crystal structure is shown in the right inset. An in-depth model of the AtEPSPS active site in the closed conformation (panel B) was obtained using the AlphaFold [46] prediction of AtEPSPS (https://alphafold.ebi.ac.uk/entry/P05466) that is in the closed conformation (AtEPSPS-AF, gray with black labels). AtEPSPS-AF was superposed onto the 1g6s structure (yellow with purple labels). (For interpretation of the references to color in this figure legend, the reader is referred to the web version of this article.)

The active site of AtEPSPS has been mapped by a superposition with the structure of EPSPS from *E. coli* (EcEPSPS, Uniprot ID: P0A6D3) in complex with S3P and glyphosate (PDB ID: 1g6s [45], Fig. 4). Due to the different state (open *vs* closed), individual domains of AtEPSPS were used to reveal interacting residues. The catalytic venue is located in a deep cleft that is formed at the interdomain interface (Fig. 4A). N-ends of α-helices α10-14 (terminal domain) and α1, α2, α5, and α7-9 (central domain) point toward the active site. Their helical dipoles increase the positive charge within the interdomain cleft that has evolved to attract negatively charged S3P and PEP, as well as glyphosate (Fig. 4A). This superposition simultaneously revealed S3P and glyphosate binding poses that likely occur before EPSPS closing (Fig. 4A). According to this model, the terminal domain binds the carboxyl moiety of glyphosate and both hydroxyls of S3P. The central domain, whose active site surface has a strong positive charge, interacts with the phosphate of glyphosate and with carboxylate and phosphate of S3P.

Interestingly, the chloride anion found in our crystal structure does not mimic either of the ligand carboxylates or phosphates (Fig. 4A, right inset). Instead, it interacts with the N-end of the α1 helix, with the backbone amides of Lys99 and Ser100 pointing towards Cl^-^ (3.2–3.9 Å distance, not shown). This Cl^-^ position marks a viable site for anionic moieties of future EPSPS inhibitors.

Due to the lack of an experimental structure of AtEPSPS in the closed form, we used the recently released model from AlphaFold prediction [46] (https://alphafold.ebi.ac.uk/entry/P05466), referred to as AtEPSPS-AF, to envisage the protein-ligands interactions (Fig. 4B**)**. It is apparent that the 1g6s structure had a big impact on the AlphaFold prediction as the RMSD between 400 pruned Cα atom pairs is 0.75 Å (across all 426 pairs: 1.534). Strict conservation of the residues binding S3P and glyphosate suggests that selectivity of plant *vs* bacterial EPSPS inhibitors will be difficult to reach if only these regions are targeted.

### 2.4 Comparison with *E. coli* EPSP synthase and other homologs

**Fig. 5:**
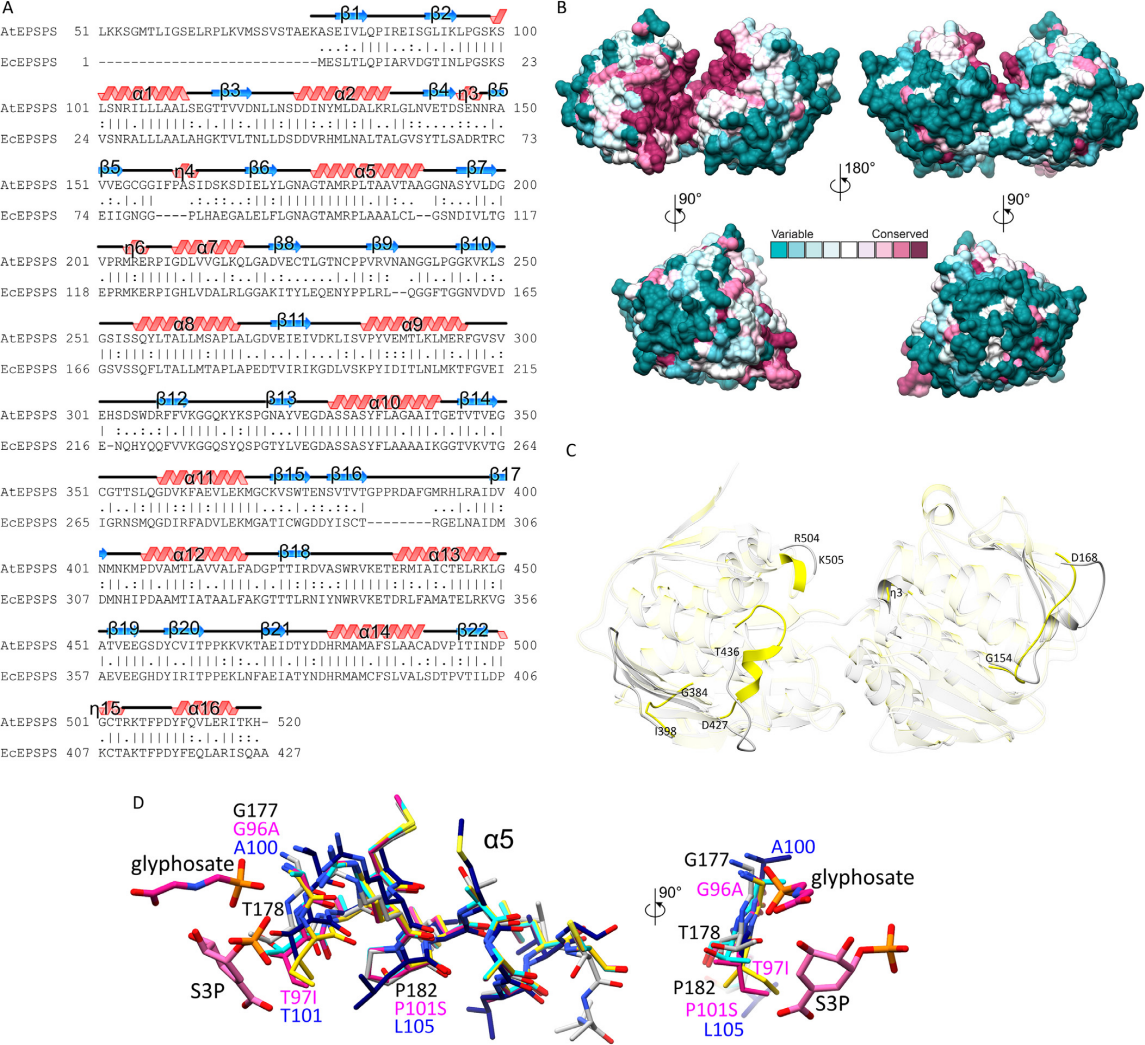
Comparison of AtEPSPS with its homologues from other species. Panel A presents the sequence alignment of AtEPSPS with EcEPSPS; secondary structure elements are shown for this work structure. Residue conservation in EPSPS homologs sharing between 35% and 95% sequence identity to AtEPSPS is shown in panel B, color-coded according to the key. Panel C presents the structural comparison of this work AtEPSPS (gray) structure with EcEPSPS (yellow, PDB ID 1g6s [45]). Due to the different conformation in the 1g6s structure, the terminal and central domains were superposed and compared independently. Fragments that overlap well are semitransparent to highlight the differences. Residue numbering is given for AtEPSPS. Superposition of the structures of glyphosate insensitive EPSPS variants onto the AtEPSPS structure is shown in panel D. The central domains were superposed but only the α5 helix (in two views) is visualized for clarity. Residue substitutions tested in EcEPSPS are labeled in magenta while corresponding positions in AtEPSPS and CP4 EPSPS are black and navy blue, respectively. The compared structures of EcEPSPS are: G96A mutant (cyan, PDB ID: 1mi4, [51]); T97I (pink, 3fjz, [50]); T97I/P101S (yellow, 3fko, [50]); CP4 EPSPS (navy blue, 2gg6, [52]). Glyphosate and S3P originate from the 3fjz structure. The view in panel D (left) is similar to that in C. (For interpretation of the references to color in this figure legend, the reader is referred to the web version of this article.)

AtEPSPS and EcEPSPS share 45% overall sequence identity and 59% overall sequence similarity; the alignment is presented in Fig. 5A. To gain more insights into residue conservation in EPSPS enzymes, we analyzed 500 sequences that sampled 4595 homologous entries from the Uniref90 database using Consurf [47], [48]. The result, mapped on the surface of AtEPSPS, revealed that only residues in or near the cleft between the domains (where the active site is located, see below) are highly conserved, while the rest of the protein surface is variable (Fig. 5B).

Next, the comparative analysis of AtEPSPS structure with EcEPSPS was performed using atomic coordinates retrieved from the PDB ID 1g6s entry [45]. Due to the different conformation in the 1g6s structure, both domains (terminal and central) were superposed and compared independently (Fig. 5C). Residues 1–20 and 242–427 of EcEPSPS were included in the terminal domain, whereas the central domain contained the remaining residues 21–241. Isolated domains superpose well, which was not true for the entire structure. In particular, the superposition of terminal domains of AtEPSPS and EcEPSPS revealed the RMSD value of 0.81 Å (169 out of 205 Cα pairs were included based on a 2-Å distance cutoff). The central domains are more variable, given the RMSD value of 0.97 Å (187 of 221 Cα pairs). The key differences occur in regions outside the domain cores that have well-defined secondary structure.

Lying at the edge of the active site cleft, the loop between β18 and α13 of AtEPSPS (residues Asp427-Thr436) is remarkably different compared to the corresponding fragment in EcEPSPS. In EcEPSPS, the fragment (residues 333–343) contains a helix (335–339) (PDB ID 1g6s). It seems unlikely that the ligand binding drives the loop-to-helix transition as the helix is also present in the unliganded structures of *Streptomyces sviceus* EPSPS (PDB ID: 7m0o [49]) and in the Cα-trace of EcEPSPS (1eps). Furthermore, also at the cleft interface of the terminal domain, the helix of EcEPSPS that is the counterpart of the η15 helix in AtEPSPS is longer by two residues (Ala410-Lys411). The corresponding fragment in AtEPSPS (Arg504-Lys505) does not extend the η15 helix.

Other significant differences between AtEPSPS and EcEPSPS occur in regions further from the active site. Two are evident at opposite poles of the protein. The fragment between β5 and β6 (residues 154–168) is four residues longer in AtEPSPS and contains a 3_10_ helix (η4). On the other side, the counterpart of the loop Gly384-Ile398 of AtEPSPS is eight residues shorter in EcEPSPS (Arg298-Ile304). In AtEPSPS, this long loop forms a lid-like structure that shields the C-end of the α10 helix from the solvent. Furthermore, EcEPSPS lacks the η3 3_10_ helix counterpart. Altogether, these results suggest that in order to achieve selectivity of novel herbicides *vs* antibiotics, areas outside the active site cleft should be exploited to bind fragments of inhibitors. Such selectivity-providing moiety could then be covalently linked to, e.g., a substrate or transition state analog moiety, which would bind at the active site. The cumulative effect would then ensure both high affinity and selectivity.

Both AtEPSPS and EcEPSPS are sensitive to inhibition by glyphosate [50], [51]. Mutated variants of EcEPSPS which confer glyphosate insensitivity have been investigated structurally and those results have been instrumental in understanding glyphosate resistance. For instance, the G96A mutation in EcEPSPS shifts IC_50_ from 10 µM to above 10 mM [51]. Substitution of the subsequent residue, Thr97 in EcEPSPS also desensitizes the enzyme to glyphosate, as revealed by the IC_50_ of 330 µM reported for the T97I mutant [50]. However, this mutation decreases the affinity to PEP, which is restored in the T97I/P101S double mutant that exhibits IC_50_ to glyphosate of 6.6 mM [50]. Thr97 and Pro101 correspond to Thr178 and Pro182 in AtEPSPS, respectively. All three substitutions (corresponding to Gly177, Thr178, and Pro182 in AtEPSPS) occur within the α5 helix positioned in the core of the central domain (Fig. 5D).

Glyphosate-sensitive AtEPSPS and EcEPSPS belong to the so-called Class I of EPSPS enzymes that exist in all plants and many bacteria. However, genomes of some bacteria encode EPSPS of Class II, which are naturally resistant to glyphosate. The enzyme from *Agrobacterium* sp. strain CP4 is one such example, exhibiting IC_50_ to glyphosate of 11 mM [52]. CP4 EPSPS can still bind glyphosate, but the herbicide conformation is then more condensed compared to the binding mode in EcEPSPS. Interestingly, the CP4 EPSPS sensitivity to glyphosate is restored in A100G mutant; Ala100 is equivalent to Gly177 of AtEPSPS and Gly96 of EcEPSPS. Overall, it is interesting that glyphosate insensitivity can be attributed to minute differences which, by design, do not to prevent specific H-bonding. With that in mind, the first structure of a plant EPSPS enzyme has the potential to be used as an improved scaffold in designing or predicting glyphosate resistance.

### 2.5 Dynamics of AtEPSPS

**Fig. 6:**
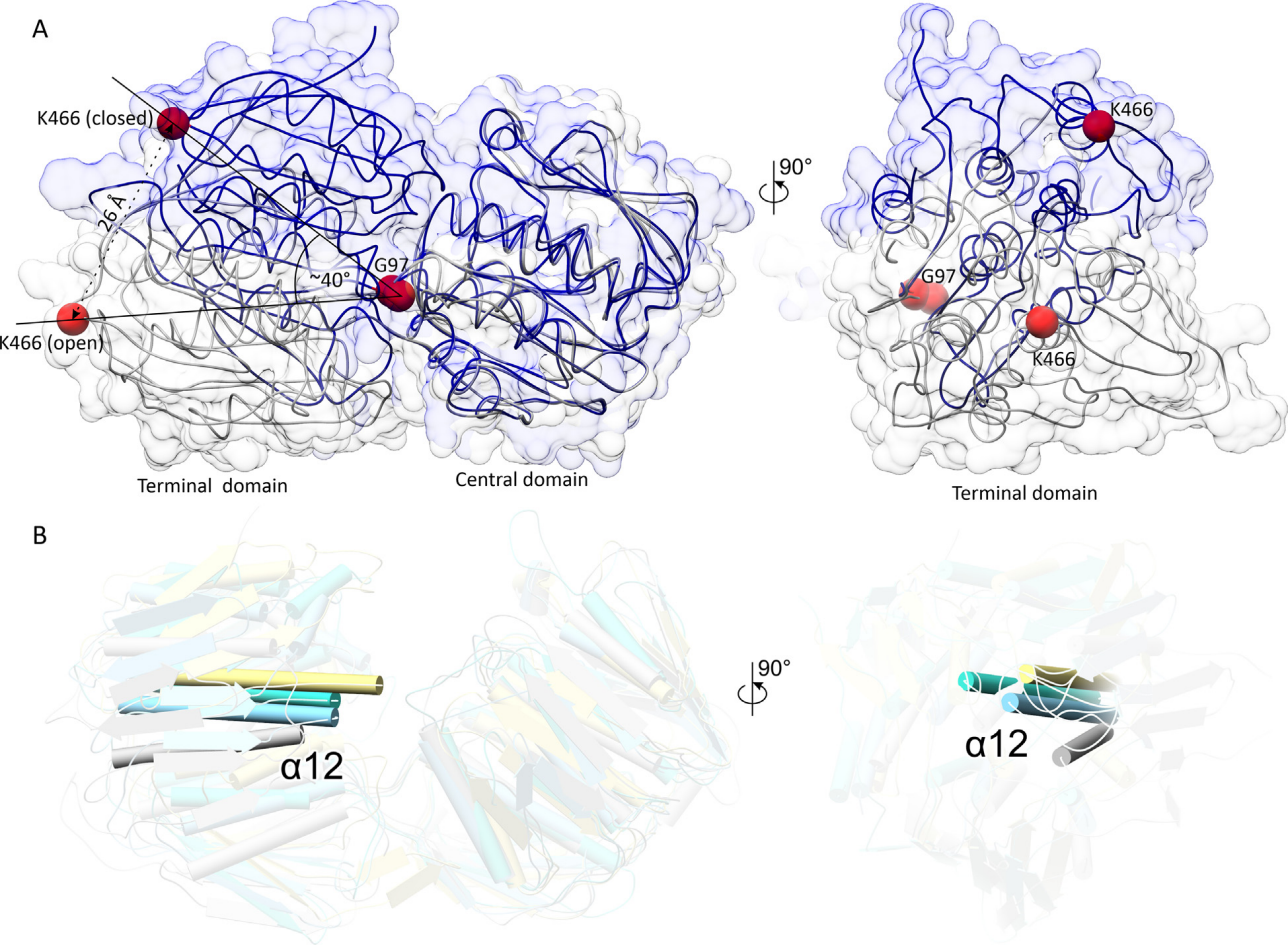
Dynamics of AtEPSPS. In panel A, the central domains of this work structure (open, gray) and AtEPSPS-AF (closed, dark blue) are superposed to reveal the large-scale conformational rearrangements. As the positioning of Gly97 (at the interdomain boundary) changes little, the angle is defined between the Cα atoms of Lys466 (open), Gly97, and Lys466 (closed). The shift of Lys466 by 26 Å means that the domains tilt by as much as 40°. Panel B illustrates the superposition (by the central domains) of four EPSPS structures in the open (unliganded) state, AtEPSPS (this work, gray); 7m0o (*S. sviceus*, [49], light blue), 2bjb (*M. tuberculosis*, unpublished, turquoise), and 2gg4 (*Agrobacterium* sp. *CP4*, [52], khaki). For clarity, the protein chains are semitransparent, except for the α12 helix and its equivalents. (For interpretation of the references to color in this figure legend, the reader is referred to the web version of this article.)

As stated above, the AlphaFold model of AtEPSPS is in the closed conformation – similar to that of EcEPSPS in the 1g6s structure. To investigate the dynamics of AtEPSPS, we superposed our crystal structure with AtEPSPS-AF at their central domains (Fig. 6A). It must be noted that while the AlphaFold model can be treated as the extremely closed conformation, it is possible that AtEPSPS can open beyond the conformation seen in our crystal structure. Nonetheless, in this comparison, the movement of domains with respect to one another is already ∼ 40°, as determined by the angle between the Cα atoms of Lys466(closed)-Gly97-Lys466(open). The domain movement corresponds to a shift in the Cα position of Lys466 by 26 Å. To our knowledge, the dynamics of EPSPS have not been exploited in the search for novel inhibitors. This is a lost opportunity as inhibitors, or at least their moieties, binding near or at the inter-domain hinge region could prevent the enzyme from closing, which is essential to form the functional active site. As the hinge region is variable across species (Fig. 5B), such an approach would provide another way to reach selectivity.

We were also intrigued whether a similar (open) conformation structure has been previously shown for any EPSPS enzyme. Notably, most of the EPSPS structures in the PDB are in the closed form. We deployed PDB-Fold [53] to identify examples in the open state. The search across the entire PDB revealed that the closest three structures are 7m0o (RMSD = 2.38 Å; *S. sviceus*; [49]), 2bjb (RMSD = 3.31; *Mycobacterium tuberculosis*; unpublished), and 2gg4 (RMSD = 3.76; *Agrobacterium* sp. CP4; [52]). The large RMSD values indicate significantly different conformations; all three structures are unliganded. When the central domains are superposed, positions of the terminal domains vary significantly (even more than 10 Å; Fig. 6B). Furthermore, these positions do not seem to be on the common trajectory that would allow the enzyme to close. This observation suggests that the hinge of EPSPS enzymes may function similar to a ball-joint, allowing for domain movement in more than one plane.

### 2.6 Virtual screening of fragment-like molecules

**Fig. 7:**
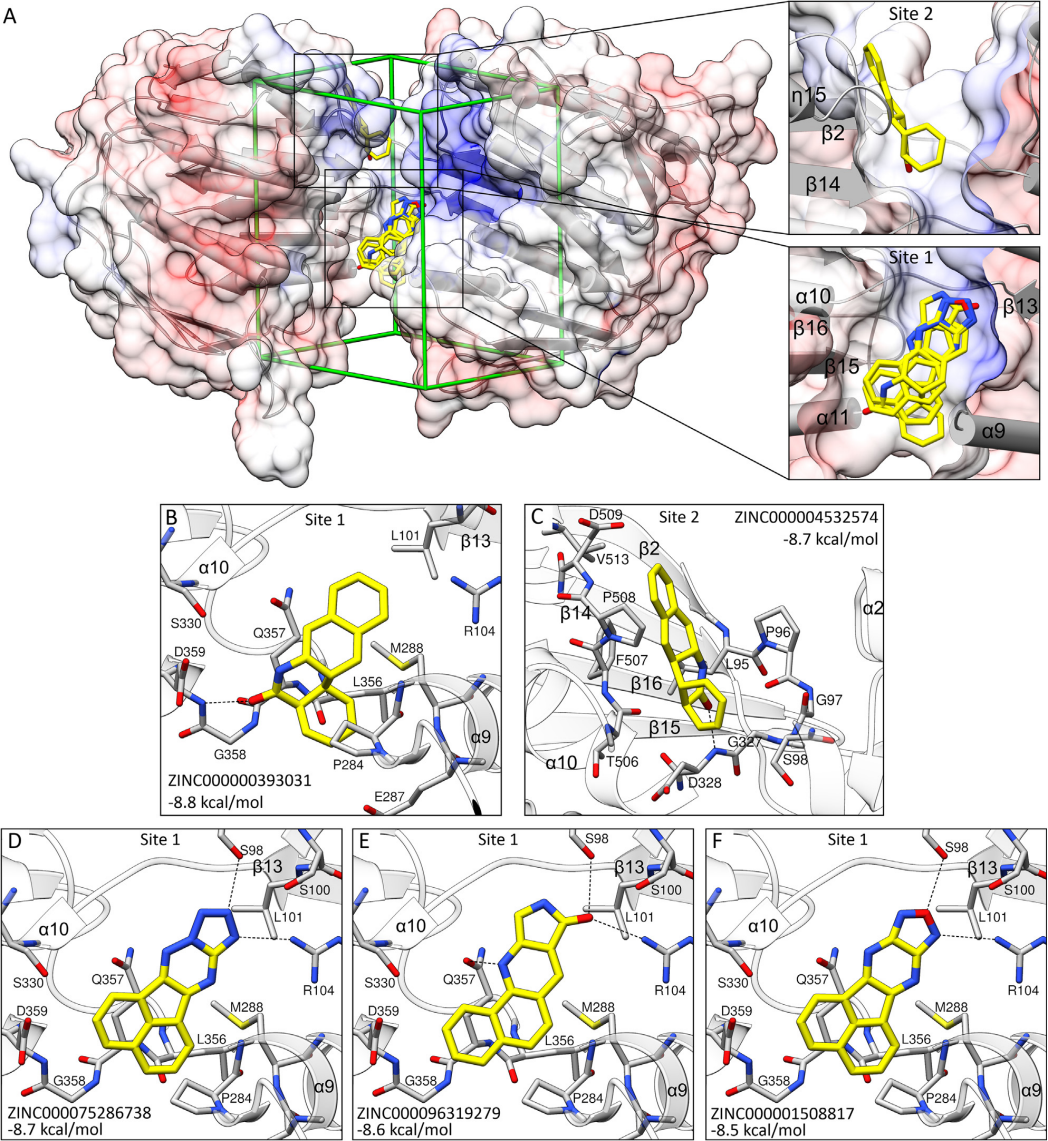
Virtual screening of the fragment library from the ZINC database [54] by docking in Autodock Vina to the open AtEPSPS structure. Edges of the search box are shown in panel A as green sticks; the view is rotated 30° (front-downwards) around the *x* axis compared to Fig. 4A. Insets present close-up views over the two discussed binding sites. Panels B-F present five best scoring hits (yellow sticks) ordered by the estimated binding energy, given in kcal/mol in each panel together with the ZINC ID. (For interpretation of the references to color in this figure legend, the reader is referred to the web version of this article.)

We also performed *in silico* docking of over 800 000 “fragment-like” molecules retrieved from the ZINC database [54]. The search box spanned the entire cleft of this work crystal structure (Fig. 7A). We used this approach to produce two types of results: (i) pinpoint sites in EPSPS structure that are good binders of small molecules and (ii) identify chemical moieties binding at those sites.

The five best scoring hits had the calculated binding energy between −8.8 and −8.5 kcal/mol (Fig. 7B-F). They all bound at the interdomain hinge, ∼5 Å deeper into the active-site cleft than either substrate or glyphosate. There are two sites that appear as particularly good binders of the fragments. One of those sites (Site 1) is located near the niche-pointing ends of α10, α11, β15, β16 from one side and α9 and β13 from the other (Fig. 7A). Universal features of the predicted ligand poses are H-bonds formed either by the backbone amide of Asp359 or side chains of Ser98 and Arg104. Aromatic rings of the ligands fit well into the hydrophobic environment created by Leu101, Pro284, Leu356, and Met 288; the latter may form S-aromatic interactions. The other site (Site 2) is formed near the ends of β2, β14 and η15 (Fig. 7A,C). The backbone amide was involved in the hydrogen bond, whereas the hydrophobic environment was provided by Leu95, Pro96, Phe507, and Pro508 (Fig. 7C).

Many other molecules, estimated to bind with a weaker energy gain, also bound to either of these two sites. Moreover, the location of these sites in the open EPSPS structure and their “disappearance” in the closed conformation strongly suggest that binding of molecules at those locations may perturb the EPSPS dynamics and hence inhibit the enzyme activity.

## 3 Conclusions and outlook

This work presents a thorough functional and structural characterization of EPSPS from *A. thaliana*. While the active site of the EPSPS enzyme is conserved across superkingdoms, other regions, such as those surrounding the entrance to the active site cleft or the interdomain hinge, are highly variable. Two sites near the hinge of AtEPSPS were identified through virtual screening of over 800 000 molecules. Binding of small molecules at these sites would most likely interfere with closing of the enzyme that is required to rebuild the active site in each reaction cycle. Targeting of these sites in future research seems thus promising and viable to design novel EPSPS inhibitors. Such molecules, with high selectivity for plant EPSPS enzymes, could be used as next-generation herbicides to supersede glyphosate, whose future is uncertain. In this context, this work provides a reliable scaffold for computer-aided herbicide design, especially considering that the AtEPSPS structure was obtained at physiological pH.

For a couple of decades, many studies aimed at the identification, prediction or design of glyphosate insensitive EPSPS variants. For instance, very recent work by Leino and co-workers showed a comprehensive classification of the human gut microbiome in the context of glyphosate resistance [55]. These authors also present a bioinformatic tool that can predict glyphosate sensitivity from a protein sequence. Now, the experimental structure of a plant EPSPS will enable the use of machine learning to predict glyphosate sensitivity in plant species and biotypes based not only on the sequence but also on the protein structure. It must be noted that protein structure predictions, such as those generated by AlphaFold [46], are template-based. In other words, the closer the templates are to the modeled structure (e.g., from the PDB [56]), the more accurate the structure prediction will be. Moreover, as seen in EcEPSPS, even minute structural changes can result in drastically different enzyme properties, many of which cannot be deduced rationally based solely on the sequence [50]. Therefore, this work has the potential to open new horizons for the modern agriculture.

## 4 Materials and methods

### 4.1 Cloning, overexpression and purification of AtEPSPS

The AtEPSPS production was performed according to the protocol established for other plant proteins [57]. The complementary DNA (cDNA) was obtained via reverse transcription reaction on the total RNA isolated from *A. thaliana* leaves using the RNeasy Plant Mini Kit (Qiagen). The construct for the overexpression of AtEPSPS (Uniprot ID: P05466; locus At2g45300) was designed based on a comparative analysis of homologous sequences from plants and prediction of signal peptides using the TargetP 1.1 server [58], [59]. The final polypeptide started from genuine Lys77, preceded by the Ser-Asn-Ala fragment introduced from the pMCSG68 vector. Accordingly, the primers (Forward: TACTTCCAATCCAATGCCAAAGCGTCGGAGATTGTACTTCAACC, Reverse: TTATCCACTTCCAATGTTAGTGCTTTGTGATTCTTTCAAGTACTTGGAA) were used to amplify the desired sequence by PCR. Next, the ligase-independent cloning method [60] was employed to create the expression plasmid based on the pMCSG68 vector backbone (Midwest Center for Structural Genomics). DNA sequencing confirmed the insert correctness.

BL21 Gold *E. coli* cells (Agilent Technologies), transformed with the expression plasmid, were cultured (at 190 rpm at 37 °C) in LB media supplemented with 150 μg mL^−1^ ampicillin. When the A_600_ reached 1.0, they were chilled to 18 °C, isopropyl-D-thiogalactopyranoside (IPTG) was added at a final concentration of 0.5 mM, and overexpression was carried out for 18 h. The cell pellet from the 4 L culture was centrifuged (3500 × *g*, 30 min, 4 °C) and resuspended in 35 mL of binding buffer [50 mM Hepes-NaOH pH 7.5; 500 mM NaCl; 20 mM imidazole; 1 mM tris(2-carboxyethyl)phosphine (TCEP)] and stored at −80 °C.

The cells were lysed by sonication in an ice/water bath using bursts (4 s ON and 26 s OFF) for 5 min of the probe “ON” time. The cell debris was removed by centrifugation at 25,000 × g for 30 min at 4 °C. The supernatant was mixed with 4 mL of HisTrap HP resin (GE Healthcare) and transferred to a 50 mL column plugged into a vacuum pump-VacMan setup (Promega). The resin-bound AtEPSPS was washed six times with 40 mL of the binding buffer and eluted with 20 mL of elution buffer (50 mM Hepes-NaOH pH 7.5; 500 mM NaCl; 400 mM imidazole; 1 mM TCEP). The His_6_-tag was cleaved with TEV protease (at a final concentration of 0.1 mg mL^−1^) overnight during simultaneous dialysis (at 4 °C) to lower the imidazole concentration to 20 mM. The sample was mixed with fresh HisTrap resin and the flow-through (containing AtEPSPS) was collected. The sample was concentrated to 2.4 mL volume and applied onto a HiLoad Superdex 200 16/60 column (GE Healthcare), equilibrated with a buffer composed of 25 mM Hepes-NaOH pH 7.5, 100 mM KCl, 50 mM NaCl, and 1 mM TCEP. The entire purification procedure (for crystallization) was completed within 24 h.

### 4.2 Crystallization and diffraction data collection

AtEPSPS was concentrated using centrifugal concentrators (Millipore) to 24 mg mL^−1^ (based on A_280_ with the extinction coefficient of 32,900). The protein was incubated with glyphosate for 1 h. The crystals were grown by vapor diffusion method in sitting drops containing 2 µL of the protein and 2 µL of the reservoir solution composed of 75% of the Index (Hampton Research) G12 (0.2 M MgCl_2_ 0.1 M HEPES pH 7.5 25% w/v Polyethylene glycol 3,350) and 25% water. The crystals were cryoprotected with the Index G12 condition supplemented with 20% of ethylene glycol and 10 mM glyphosate and flash-frozen in liquid nitrogen. Data were collected at the 22-ID beamline at the Advanced Photon Source, Argonne, USA. The diffraction images were processed with *XDS* [61]. The statistics of the data collection and processing are summarized in Table 1.

**Table 1:** Data collection and refinement statistics. Values in parentheses correspond to the highest resolution shell.

	AtEPSPS-Nt77
**Data collection**	
Beamline	APS 22-ID
Wavelength (Å)	1.0000
Space group	*I*422
Unit cell parameters	
*a = b, c* (Å)	108.4, 156.8
Resolution (Å)	80–1.40 (1.48–1.40)
Unique reflections	92,087 (14710)
Multiplicity	18.5 (18.7)
Completeness (%)	99.9 (99.7)
*R*_merge_^a^(%)	6.6 (140.7)
<*I*/σ(*I)*>	21.8 (2.1)
**Refinement**	
*R*_free_ reflections	1012
No. of atoms (non-H)	
protein	3345
ligands	2
solvent	478
*R*_work_/*R*_free_ (%)	15.2 / 19.0
Average B-factor (Å^2^)	
protein	31.5
ligands	51.5
solvent	44.7
RMSD from ideal geometry	
bond lengths (Å)	0.01
bond angles (^o^)	1.0
Ramachandran statistics (%)	
favored	97.3
allowed	2.5
outliers	0.2
PDB ID	7pxy

*^a^ R*_meas_ = redundancy independent R-factor [72].

^b^ Value for subunit A.

### 4.3 Determination and refinement of the crystal structures

The AtEPSPS crystal structure was solved by molecular replacement with *PHASER* [62] using separate domains of its closest homolog (56% sequence identity) in the PDB, *Vibrio cholerae* EPSPS (PDB D: 3nvs, unpublished). The initial model was built with *Phenix*.*AutoBuild* [63] and was placed inside the unit cell with the *ACHESYM* server [64]. *COOT* [65] was used for manual fitting in the electron density maps between rounds of model refinement in *Phenix.refine* [66]. The atomic displacement parameters were refined anisotropically for all non-H atoms. The refinement statistics are listed in Table 1.

### 4.4 Enzyme assay

For kinetic characterization, bacterial pellets from IPTG-induced cultures were transferred into a pre-cooled mortar and ground with alumina (2 g [g cells]^-1^) until a fine paste was obtained. All subsequent operations were carried out at 0 to 4 °C. The homogenate was resuspended with 25 mL g^−1^ of extraction buffer (50 mM Hepes-KOH buffer, pH 7.4, containing 5% [v/v] glycerol, 200 mM NaCl, 0.5 mM dithiothreitol, 0.5 mM EDTA and 10 µM ammonium molybdate), and clarified for 10 min at 14,000 × *g*. The extract was loaded at a constant flow of 10 mL h^−1^ onto a His-SpinTrap^TM^ Nickel Sepharose Gel column (GE Healthcare, Little Chalfont, UK; 0.1 mL bed volume) equilibrated with extraction buffer. Following extensive washing, elution was achieved by the same buffer containing 50 mM imidazole while collecting 1.5-mL fractions.

EPSP synthase activity was measured in the forward, physiological direction by quantifying the inorganic phosphate release using the malachite green dye assay method as described, with minor modifications [21]. The reaction assay contained 50 mM Hepes-KOH, pH 7.4, 1 mM S3P, 1 mM PEP and a limiting amount of enzyme (5 to 10 pkat) in a final volume of 30 μL. Samples were incubated in wells of a 96-well microplate at 35 °C for up to 5 min, then the reaction was stopped by the addition of 200 μL of the malachite green-molybdate-acid colorimetric solution followed, after 1 min, by 20 μL of 34% (w/v) Na citrate. After a further 20 min at room temperature, absorption at 660 nm was measured against exact blanks (in which S3P had been omitted) using a Ledetect plate reader (Labexim, Lengau, Austria) equipped with a LED plugin. The activity was calculated from the initial linear rate on the basis of an extinction coefficient for phosphate ranging from 80,000 to 90,000 M^−1^ cm^−1^, evaluated experimentally for each batch of colorimetric solution. For kinetic analysis, the invariable substrates were fixed at 1 mM, whereas the variable substrate ranged from 100 to 1000 μM. Apparent affinity constants (K_M_) and maximal rates (V_max_), and their confidence limits, were computed by linear regression of Lineweaver-Burk plots of data.

The ammonium salt of S3P was purified by anion-exchange chromatography from the growth medium of *Klebsiella pneumoniae*, strain ATCC 25597, and quantified by RP-HPLC following the treatment with alkaline phosphatase, as described previously [41]. Glyphosate was purchased from Sigma (P5671).

Each sample was assayed in triplicate, and each experiment was repeated three times with independent enzyme preparations. Linear (enzyme activity assay, K_M_, K_I_, V_max_) and non-linear (glyphosate IC_50_) regression analyses were computed by using Prism 6 (version 6.03, GraphPad Software, Inc., USA).

### 4.5 Virtual screening

The library of 808,202 molecules was downloaded from the ZINC database [54] in May 2020. Docking was performed in *AutoDock Vina* [67] using custom made Python scripts to manage the process, with the exhaustiveness = 8. This work crystal structure, prepared with the UCSF *Chimera* DockPrep tool [68], was used as the receptor. The search box was centered at *x*  = 16, *y* = 35, *z* = 20, with the dimensions of 20, 27, 30.5 Å, respectively. The results were scored based on the calculated binding energy.

### 4.6 Other software used

Molecular illustrations were created with UCSF *Chimera* [68], which also served for calculations of the RMSD values for Cα atom pairs within the default 2-Å radius. RMSD values for the whole PDB search were taken from PDB-Fold [53]. The surface conservation was analyzed with *ConSurf* [47] based on 500 sequences that sampled homologs from Uniprot [69] between 35 and 95% sequence identity to AtEPSPS. The distribution of the surface electrostatic potential was calculated using *PDB2PQR* and *APBS* servers [70], [71].

## Accession numbers

PDB ID: Crystal Structure of *Arabidopsis thaliana* 5-*enol*-Pyruvyl-Shikimate-3-Phosphate Synthase, 7pxy.

## Author statement

MR designed the project, produced and crystallized the protein and solved the structures; GF performed kinetic analysis. Both authors wrote the manuscript.

## Declaration of Competing Interest

The authors declare that they have no known competing financial interests or personal relationships that could have appeared to influence the work reported in this paper.
